# Multifactorial Evaluation following Cytoreductive Surgery for Malignant Pleural Mesothelioma in Patients with High Symptom-Burden

**DOI:** 10.3390/jcm11216418

**Published:** 2022-10-29

**Authors:** Riccardo Tajè, Roberto Fiorito, Alexandro Patirelis, Valentina Marziali, Vincenzo Ambrogi

**Affiliations:** 1 Thoracic Surgery Department, Tor Vergata University Polyclinic, 00133 Rome, Italy; 2 Department of Biomedicine and Prevention, Tor Vergata University, 00133 Rome, Italy

**Keywords:** malignant pleural mesothelioma, quality of life, pleurectomy/decortication, extrapleural pneumonectomy, symptom control, symptom palliation, pleural cancer

## Abstract

Mesothelioma has a scant prognosis and a great impact on symptoms and the quality of life. Pleurectomy/decortication and extrapleural pneumonectomy are the two cytoreductive surgical strategies, with different invasiveness, but achieving similar oncological results. Hereafter, the two surgical procedures effects on symptoms and the quality of life are compared in a high symptom-burden population. Between 2003 and 2017, 55 consecutive patients underwent pleurectomy/decortication (*n* = 26) or extrapleural pneumonectomy (*n* = 29), both followed by adjuvant chemo-radiotherapy. Cardio-pulmonary function, symptoms and the quality of life (Short-Form-36 and St.George’s questionnaires) were evaluated pre- and 3-, 6-, 12- and 24-months postoperatively. Extrapleural pneumonectomy demonstrated lower pain at 12 months but a higher decrement of forced vital capacity at 24 months than pleurectomy/decortication. Both procedures revealed a 3-months improvement of many symptoms and the quality of life determinants. Improvement in physical, social and pain-related measured parameters lasted for a longer time-spawn in the extrapleural pneumonectomy group. No differences were found in chemotherapy compliance and survival between groups. Age-at-presentation (*p* = 0.02) and non-epitheliod histology (*p* = 0.10) were the only significant prognosticators. Surgery, despite poor survival results, improved symptoms and the quality of life in patients with mesothelioma with high symptom-burden at diagnosis. Therefore, extrapleural pneumonectomy demonstrated the most durable effects.

## 1. Introduction

Malignant pleural mesothelioma is a rare and highly aggressive tumor with a poor prognosis [1,2]. Only a small number of patients present at diagnosis with a surgically resectable disease. Surgical management of this highly morbid disease is strongly suggested even if the best surgical approach is still debated [3]. Extrapleural pneumonectomy (EPP) and pleurectomy/decortication (PD) are the two cytoreductive surgical procedures. The former is an invasive procedure with a significant rate of complications and perioperative morbidity and mortality, while the latter represents an alternative lung-sparing surgery to adopt in the case of poor residual lung function or significant comorbidity. Despite the differences in surgical invasiveness, both the procedures are aimed at removing visible and palpable disease. Thus, there appears to be no role for surgery alone in the management of this aggressive disease [4]. Encouraging results have been obtained in selected patients at early stages with multimodal approaches including surgery and adjuvant chemo-radiotherapy, but survival and recurrence rates are still heterogeneous [4,5,6,7,8,9].

Given life expectancy is generally low, the quality of life assumes a leading role. The impact and duration on the effects of surgery on patients’ quality of life seems to be morally crucial. An impaired quality of life has been associated with early chemotherapy discontinuation and reduced overall survival [10,11]. Therefore, both the limited life expectancy and the need to promptly initiate treatment make the quality of life after surgery a relevant issue in the treatment of malignant pleural mesothelioma. Surgery was demonstrated to improve symptoms and the quality of life, especially in patients with a high pre-operative symptom-burden [12]. Thus, evaluating the durability of surgical effects on symptoms between the two surgical techniques can play a key role in the surgical management choice.

In this retrospective study, we compared the effect of pleurectomy/decortication and extrapleural pneumonectomy on the symptoms and the quality of life in patients affected by malignant pleural mesothelioma with a preoperative high symptom-burden at fixed time point. The durability of the effects of surgery on respiratory function, the quality of life, and symptom control between the two procedures, in the two years after surgery, have been compared.

## 2. Materials and Methods

### 2.1. Patients Selection and Preoperative Assessment

From 2003 to 2017 inclusive, 55 consecutive patients, 49 men and 6 women (median age 61.8 ± 10.0 years), underwent intentional extended pleurectomy/decortication (26/55; 47.3%) or extrapleural pneumonectomy (29/55; 52.7%). Clinical and pathological features of the study group are summarized in Table 1.

All patients had undergone total body computed tomography (CT) scan. Mediastinoscopy was performed whenever CT evidenced enlarged upper mediastinal lymph nodes. Patients were staged according to the TNM-staging system by the International Mesothelioma Interest Group [13,14]. All the patients underwent pathologic characterization of the disease through video-assisted thoracic surgery about 30 days before cytoreductive surgery. In the case of patients presenting at diagnoses with pleural effusion, thoracoscopic pleural biopsies and a cytological examination of the pleural effusion were obtained to achieve a differential diagnosis with secondary pleural malignances about 30 days before cytoreductive surgery. For these reasons, all the patients arrived at cytoreductive surgery with no pleural effusion and with a satisfactory re-expansion of the involved lung.

Demographic and perioperative characteristics of the enrolled patients were collected in a prospectively organized database with standardized entries. Data on long-term outcomes including symptoms, the quality of life, and survival, were determined the day before cytoreductive surgery and 3-, 6-, 12- and 24-months postoperatively. A fully informed consent in order to manage clinical data for scientific purposes was asked for and signed by all patients.

### 2.2. Surgical Technique

Extended pleurectomy/decortication involves the complete resection of both visceral and parietal pleura including, whenever necessary, pericardial and/or diaphragmatic resection after an intraoperative evaluation [2,3]. Extrapleural pneumonectomy implies en-bloc resection of parietal pleura, lung, homolateral hemidiaphragm and pericardium with pericardial and diaphragmatic reconstruction. We performed the operations through an extended posterolateral thoracotomy in the sixth intercostal space associating an additional counterincision in the eighth space. In both pleurectomy/decortication and extrapleural pneumonectomy, all mediastinal lymph nodes were routinely resected in order to allow an accurate surgical staging of the disease.

Inclusion and exclusion criteria for either pleurectomy/decortication or extrapleural pneumonectomy was made according to medical and surgical criteria listed in Table 2. In summary, if the lung was involved by the neoplasm, pleurectomy/decortication was considered feasible whether the resection of the neoplastic parenchymal localization could be accomplished with a non-anatomical parenchymal resection. Differently, extrapleural pneumonectomy was performed.

### 2.3. Chemotherapy and Radiation Therapy Regimens

Surgical procedure was followed by adjuvant chemotherapy in 40/55 cases (72.8%). Twelve patients received neoadjuvant chemotherapy (12/55; 21.8%) and three patients both neoadjuvant and adjuvant chemotherapy (3/55; 5.5%). Chemotherapy regimen usually consisted of four to six cycles of cisplatin (100 mg/m^2^) given at day 15, and gemcitabine (1 g/m^2^) administered on days 1, 8, and 15. Treatment was repeated every 4 weeks. Cisplatin was infused over 1 h after intravenous administration of 2000 mL saline plus potassium chloride. Gemcitabine was administered as a 30 min intravenous infusion diluted with 250 mL of saline. Pemetrexed replaced gemcitabine as it has become the standard of care in malignant pleural mesothelioma management [3]. A combined adjuvant strategy using Pemetrexed (500 mg/m^2^) and Cisplatin (75 mg/m^2^) was administered on day 1. Treatment was repeated every 3 weeks. Bevacizumab and other immune checkpoints inhibitors were not used at the beginning of the study period. To avoid bias in the quality of life and symptoms parameters, patients undergoing immunotherapy as adjuvant therapy have been excluded from this study. Treatment was interrupted because of disease progression or intolerable toxicity. Adjuvant chemotherapy was started between 4 and 10 weeks after surgery depending on the postoperative recovery.

Radiation therapy usually started after chemotherapy. External beam radiotherapy was delivered with an energy ranging from 4 to 15 MV. The total radiation doses to the hemithorax and mediastinum were normally 30 and 40 Gy, respectively, fractioned in 1.5 Gy. A boost dose (14 Gy in 2 Gy fractions) was always delivered to areas of gross residual disease or positive resection margins, and metastatic lymph nodes.

### 2.4. Clinical Follow-Up

Information was preferably retrieved by outpatient clinic or alternatively by medical records, general practitioner, or patients’ telephone call. Cross-sectional contact for all surviving patients was routinely performed every 3 months. Since imaging studies available at the beginning of the study period were not sensitive enough to accurately diagnose early recurrence, the disease-free interval was not evaluated. Therefore, the survival duration was measured from the date of surgery to the date of the patient’s last follow-up contact or death.

### 2.5. Symptom and Quality of Life Assessment

Evaluation of acute pain was self-scored by the patients like a visual analogue scale (VAS) (0 = absent, to 10 = most severe imaginable pain) during the study at timed intervals [15].

Dyspnea was rated after clinical examination according to the modified Medical Research Council score (0 = absent to 4 = maximal dyspnea) [16]. Exercise tolerance was assessed with the standard 6 min walk test. Performance status was scored according to the Karnofsky index (10% = worst impairment to 100% = no impairment) [17]. The Karnofsky index was elected as the performance status index as it was impossible to retrieve data about the Eastern Cooperative Oncology Group for all the patients enrolled in the study.

The quality of life was assessed using the Medical Outcomes Study Short Form 36-item^®^ (SF-36) [18,19] and the St. George’s Respiratory Questionnaire^®^ (SGRQ) [20,21]. SF-36 consists of 36 multiple-choice questions covering eight health concepts: physical functioning, social functioning, physical role, emotional role, vitality, body pain, mental health, and general health perception (best score = 100, worst = 0). To simplify the evaluation, the Physical Component Summary, and the Mental Component Summary (Health Assessment Lab., New England Medical Center. Boston, 1994) were also used.

SGRQ quantifies the impact of chronic airflow limitation on health and well-being. It contains 76 items organized into three sections investigating symptoms, activity, and the impact on mood of these limitations (best score = 0, worst = 100).

The durability of the effects of surgery on symptoms and the quality of life was assessed. The results obtained at each time spawn for each measured parameter have been compared with the preoperative value. Surgery was considered to affect symptoms or the quality of life until a significant difference from the preoperative values could be retrieved. At the time point when no differences could be found, the value was considered to be returned to the pre-operative value. Therefore, the durability of the effect of surgery on that symptom or the quality of life parameter was considered expired.

Questionnaire administration was approved by Institutional Ethical and Review Board of the single institutions. For ethical reasons, questionnaires were no longer administered in the case of evident neoplasm progression.

### 2.6. Objectives

The primary objective of this retrospective study was to compare the effects of pleurectomy/decortication and extrapleural pneumonectomy on symptom control and the quality of life at fixed post-operative times. Secondary outcomes were to evaluate differences between pleurectomy/decortication and extrapleural pneumonectomy on the durability of the effects of these surgical procedures in symptoms and the quality of life determinants. Finally, differences in chemo/radiotherapy administration and overall survival between the two groups were assessed.

### 2.7. Statistics

Statistical analysis was performed using the SPSS (IBM Corp. Released 2016. IBM SPSS Statistics, Version 26.0. Armonk, NY, USA: IBM Corp.). *p*-Value less than 0.05 was considered statistically significant.

Due to the small sample size, the non-parametric Wilcoxon test was used to compare the scores mean values before and after surgery. The internal consistency of the questionnaires was evaluated for global scores and specific domains using the Cronbach’s α test: considering the highest score possible for consistency equal to 1 and the lowest equal to 0. Cronbach’s α test greater than 0.7 was considered desirable.

The comparison between pleurectomy/decortication and extrapleural pneumonectomy for the scores mean values before and after surgery was performed using the non-parametric Mann-Whitney U test, due to the small number of patients.

The impact of continuous variables on survival was analyzed with the Kaplan-Meier curve and the log-rank test by dichotomizing the patients into groups according to the score above and below the median value. Factors significantly affecting survival at univariate analysis underwent a multivariate analysis by the Cox regression model.

## 3. Results

Data of the study group patients are described in Table 1. We did not experience postoperative mortality within 30 days from the procedure. Major 30-day postoperative cumulative morbidity rate was 30.9% (17/55) and included prolonged air leaks (*n* = 9), cardiac arrhythmias (*n* = 8), bleeding requiring reoperation (*n* = 2), and deep venous thrombosis (*n* = 3). Some patients had more than one cause of morbidity. The median follow-up interval was 16.5 ± 10.4 months (range 1–44 months).

### 3.1. Symptom Evaluation

Chest pain or dyspnea were the presenting symptoms in 62.5% of cases. Symptom evolution is summarized in Table 3, Table 4 and Table 5.

As depicted in Table 3, in the comparison between the two types of surgery, a statistically significant difference was observed only in pain at 12 months after surgery, with a mean two-point difference between the two procedures at visual analogue scale favoring patients undergone extrapleural pneumonectomy (*p* = 0.005), and in forced vital capacity at 2 years, with a higher mean value for patients undergone pleurectomy/decortication (*p* = 0.003).

At intergroup analysis, patients undergoing pleurectomy/decortication demonstrated a significant improvement of pain at 3 and 6 months and a decrease after that, an improvement of dyspnea and forced vital capacity up to 6 months post-operatively, followed by a return to the pre-operative values. Nonetheless, the 6 min walking test was improved up to 12 months post-operatively and decreased at 24 months towards the pre-operative values (Table 4).

As regards extrapleural pneumonectomy, pain as well as the 6 min walking test and dyspnea index were improved consistently throughout the overall follow up period. Conversely, there was a continuous and statistically significant reduction of forced vital capacity after surgery (Table 5).

The presence of pericardial or diaphragmatic prosthesis did not affect symptom evolution in any of the parameters investigated. 

### 3.2. Quality of Life Evaluation

Data regarding SF-36 SGRQ are summarized in Table 3, Table 4 and Table 5.

No significant difference was found in any follow-up for both physical and mental SF-36 domains and for SGRQ domains (Table 3). Body pain (13.7 ± 20.9 to 37.5 ± 14.6, *p* < 0.0001 for pleurectomy/decortication and 14.1 ± 20.6 to 37.9 ± 14.1, *p* < 0.001 for extrapleural pneumonectomy) and physical role functioning (42.0 ± 30.4 to 62.0 ± 28.1, *p* = 0.002 for pleurectomy/decortication and 40.2 ± 29.1 to 62.5 ± 26.8, *p* = 0.001 for extrapleural pneumonectomy) scored the greatest improvement. In the patients still alive after 6 months, only some physical domains persisted significantly greater than the baseline values. Thereafter, every domain slightly declined reaching or crossing the baseline value.

The physical component summary significantly improved (29.0 ± 11.7 to 37.1 ± 10.5, *p* = 0.001 for pleurectomy/decortication, 28.4 ± 11.3 to 37.1 ± 9.9, *p* < 0.001 for extrapleural pneumonectomy) during the first 3 months after surgery and values persisted for 6 months, whereas the mental component presented only a mild and time-limited amelioration.

As regards SGRQ, a significant improvement was found in 2 of 3 domains at 3 months postoperatively: symptoms (from 32.8 ± 26.4 to 22.2 ± 31.8, *p* = 0.042 for pleurectomy/decortication and from 32.5 ± 27.8 to 22.5 ± 31.8, *p* = 0.033 for extrapleural pneumonectomy) and activity (form 44.7 ± 29.9 to 33.0 ± 29.7, *p* = 0.029 for pleurectomy/decortication and from 43.8 ± 29.6 to 33.6 ± 30.9, *p* = 0.049 for extrapleural pneumonectomy). Thereafter, we experienced a progressive worsening of all parameters with the restoration of the preoperative status at 24 months.

No statistical difference was found with patients who required prosthetic reconstruction of the pericardium and/or diaphragm whatever the questionnaire administered.

### 3.3. Chemotherapy Compliance

The adjuvant treatment regimen was generally well-tolerated. Thirty-eight patients (69.1%) were able to receive the planned cycles of the treatment regimen. Seventeen patients did not complete the planned chemotherapy: 11 refused further treatment after two cycles and another six patients after three cycles. Radiotherapy did not produce significant morbidity unless a slight basal lung fibrosis in nine patients.

No differences could be found in chemotherapy administration between the two groups. Particularly, adjuvant chemotherapy was administered, with no delay, in 22 (21 patients had only adjuvant treatment and one patient had both neoadjuvant and adjuvant treatment) in the pleurectomy/decortication group and in 21 (19 patients had only adjuvant treatment and two patients had both neoadjuvant and adjuvant treatment) in the extrapleural pneumonectomy group (*p* = 0.44).

### 3.4. Survival Analysis

**Figure 1:**
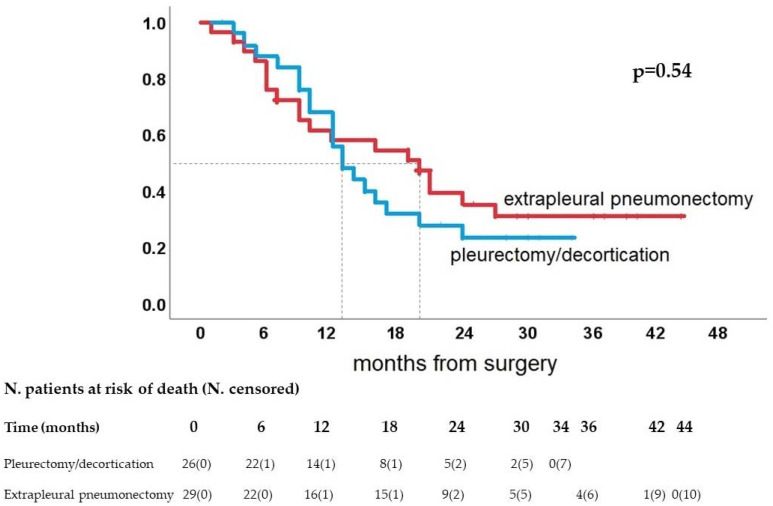
Overall survival according to type of surgery (median overall survival is indicated with dotted line).

Overall median survival was 14 months. A total amount of 37 patients (67.3%) died within 2 years from the operation. Nonepithelial histology (*p* = 0.007) and age > 65 years old (*p* = 0.001) significantly influenced the prognosis. On the contrary, the type of surgery (*p* = 0.54) (Figure 1) and any of the preoperative symptoms or the quality of life domains did not affect the prognosis. At Cox regression analysis, age > 65 years old (hazard ratio 2.91, 95% confidence interval 1.50–5.66, *p* = 0.002) and the nonepithelial histology (hazard ratio 2.41, 95% confidence interval 1.24–4.71, *p* = 0.010) were selected as the sole significant prognosticators.

## 4. Discussion

Surgery has a pivotal role in the multimodal management of malignant pleural mesothelioma. Nevertheless, the best surgical strategy in the management of this aggressive disease is currently a topic of discussion [2,3,6,9]. Pleurectomy/decortication and extrapleural pneumonectomy are the two cytoreductive surgical techniques used in mesothelioma [3].

Despite the encouraging results obtained with multimodal treatment, including surgery and chemo-radiation therapy, the disease has still a scant prognosis and significantly affects the quality of patients’ life [2,3,4,7]. The limited effects of treatment on this recalcitrant disease lets the quality of life became a top priority for these patients [22,23,24,25,26].

In this report, the effects of pleurectomy/decortication and extrapleural pneumonectomy in malignant pleural mesothelioma on symptoms and the quality of life have been compared at 3-, 6-, 12- and 24-months after surgery. At the beginning of the study period, extrapleural pneumonectomy was considered as the standard of care for surgically resectable mesothelioma, but during the years the standard of care changed towards pleurectomy/decortication. Thus, patients enrolled later in the study were more likely to undergo pleurectomy/decortication than patients enrolled at the beginning of the study period. Between the two surgical techniques, extrapleural pneumonectomy demonstrated lower pain related score, measured through the VAS scale, with the most significant difference at 12 months after surgery. Conversely, pleurectomy/decortication demonstrated to have a smaller impact on forced vital capacity when compared to extrapleural pneumonectomy with the greatest difference 24 months postoperatively.

These results are consistent with previous reports demonstrating both the transitory improvement of symptoms in patients undergoing pleurectomy/decortication and the persistent reduction in pulmonary function following extrapleural pneumonectomy. In patients undergoing pleurectomy/decortication, an initial improvement in chest pain followed by a rapid re-exacerbation within the first 6 months post-operatively was already described [27]. Indeed, pleural thickness or pleural effusion can constrict the lung triggering chest pain and discomfort [12,28]. Pleurectomy/decortication helps to re-expand the lung and may produce an initial but still relevant symptomatic relief. However, as demonstrated by Janne and Baldini [29], a loco-regional relapse may follow pleurectomy/decortication leading to a progressive worsening of chest pain, vanishing the benefits of surgery. Our results seem to differ from those reported in the MesoVATS trial that demonstrated better outcomes in the quality of life following pleurectomy/decortication compared to chemical pleurodesis [30]. However, by evaluating only the pleurectomy/decortication group in the curve proposed by Rintoul and colleagues, the quality of life mostly goes back to the baseline values at 6 months rather than improve. Under this perspective, MesoVATS trial results compare well with our findings on the quality of life, following pleurectomy/decortication. Conversely, extrapleural pneumonectomy has a significant and prolonged impact on chest pain control mainly due to the reduced rate of loco-regional relapses. However, as the lung is removed en-bloc with the pleura, a significant reduction in pulmonary functionality can follow extrapleural pneumonectomy when compared to pleurectomy/decortication [31]. This result is obviously due to the different invasiveness between the two different procedures.

**Figure 2:**
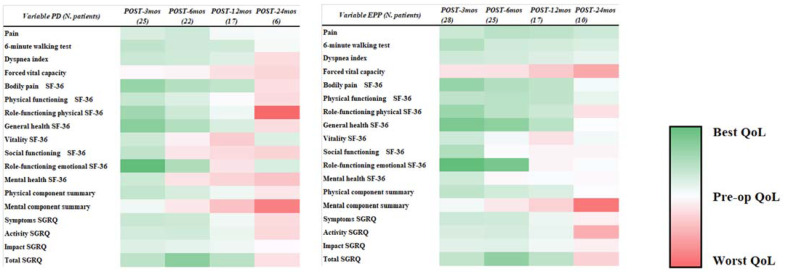
Heatmap of the durability of effects of surgery on symptoms and quality of life at fixed time spawn compared to the preoperative values. (**left**) Patients undergoing pleurectomy/decortication. (**right**) Patients undergoing extrapleural pneumonectomy. EPP: extrapleural pneumonectomy; mos: months; PD Pleurectomy decortication; Pre-op: preoperative; QoL: Quality of Life; SF-36: Short Form Health Survey 36; SGRQ: Saint George Respiratory Questionnaire.

As secondary outcomes, symptoms, and the quality of life evaluation results at fixed time points for both pleurectomy/decortication and extrapleural pneumonectomy have been independently compared with the preoperative values. Thus, the durability of the favorable and detrimental effects of surgery during the follow-up period have been compared and pictorially shown in Figure 2.

Most of the symptoms and quality of life evaluation tools demonstrated an initial improvement at 3 months after surgery when compared to the preoperative values, regardless of the surgical strategy. This seems to be in contrast with previous reports demonstrating a reduction in both symptoms and the quality of life in the early post-operative time [32,33,34]. Particularly, the MARS trial demonstrated a trend towards a poorer quality of life in patients undergoing extrapleural pneumonectomy versus patients undergoing no extrapleural pneumonectomy in the context of the trimodal therapy, especially in the early post-operative period [34]. Nevertheless, the reason behind this significant difference can be found in the preoperative symptom-burden of the enrolled patients. In our report, nearly 80% of the patients presented at surgery with a VAS higher than 5 and with a mMRC higher than 2, demonstrating severe pain and dyspnea. Therefore, the detrimental effects of surgery may have been covered up by the beneficial effects of tumor debulking, improving symptoms and the quality of life. Conversely, in the MARS trial, patients had a limited symptom-burden at randomization [34]. On symptoms control, pleurectomy/decortication and extrapleural pneumonectomy demonstrated relevant differences. Despite the previously reported larger reduction in forced vital capacity following extrapleural pneumonectomy, the perception of the respiratory effort measured through the Dyspnea Index demonstrated to be constantly improved during the 24 months follow up in patients undergoing this invasive surgical procedure. Similarly, the 6 min walking test was improved during the 24 months follow-up. Conversely, in patients undergoing pleurectomy/decortication, the Dyspnea Index was lower than the preoperative values only for the first 6 months after surgery and the 6 min walking test resulted to be improved within the first 12 months after surgery. Therefore, despite the reduction in spirometry values, extrapleural pneumonectomy was demonstrated to achieve a more durable relief of dyspnea and to improve dynamic resistance to strain in the 6 min walking test.

Despite no differences being found between the two types of surgery in the measured quality of life determinants, the differences in the durability of the effects of surgery on the quality of life may be relevant in the management of these patients. Overall, extrapleural pneumonectomy was demonstrated to improve bodily pain as measured in the SF-36 up to 12-months after surgery, while the same domain returned to the baseline preoperatory values within 6 months after pleurectomy/decortication. These results confirm the efficacy of extrapleural pneumonectomy in local control of the disease in terms of chest pain reduction, and are supported by a prolonged improvement in symptoms evaluated by the SGRQ. Pain management in patients affected by mesothelioma can be controversial and leads to uncontrolled pain despite multimodal treatment [35]. As demonstrated in previous reports, uncontrolled pain demonstrated to have a prognostic role and a detrimental impact on the quality of life [36].

Therefore, in patients with a high symptom-burden that are candidates for cytoreductive surgery, the effects of the surgical approach on symptoms and pain control durability may have a role in the management algorithm.

Pleurectomy/decortication failed to significantly enhance physical and social functioning and achieved a brief improvement of role-functioning physical only at 3 months after surgery. Differently, extrapleural pneumonectomy was proved to achieve a prolonged improvement in both physical functioning and role-physical functioning up to 6 months after surgery, as well as a 3 month improvement in social functioning. Therefore, patients undergoing extrapleural pneumonectomy achieved both prolonged control of pain and of pain impact on their lives, as well as an overall improvement of their everyday and social functioning, with a lower disease-related impact on their lives. As expected, at 24 months after surgery, both groups showed a drop in the mental component summary. Despite the efficacy of each surgical strategy in symptom control and the quality of life improvement, the trend towards the pre-operative high symptom-burden characterizes all the measured parameters. The relation between symptom-burden and mental status impairment has been previously stated in patients with advanced cancer and further enlighten the role of a mental support along with the oncological pathway [37].

When analyzed, no differences could be found in the chemo/radiotherapy compliance rate. Therefore, the surgical strategy did not affect the continuation of the multimodal approach. On the contrary, we noted an elevated compliance to chemotherapy after any kind of surgery. This is possibly due to the amelioration of perceived general health status. Indeed, patients enrolled in this study presented a high preoperative symptom-burden that was partially relieved by the surgical treatment. Therefore, the surgical-induced symptoms relief may have enhanced treatment compliance in this population.

Notably, we also found that patients undergoing diaphragmatic or pericardial reconstruction did not show any difference in the quality of life compared to the others, thus implying that demolitive surgery on advanced stages, where adequately treated, did not alter the perceived heath status.

Finally, the surgical approach was demonstrated not to have an impact on prognosis as well as symptoms and quality of life determinants. The prognostic role of symptoms and the quality of life on survival in malignant pleural mesothelioma has been previously stated [38]. Nevertheless, as the ability of symptoms and the quality of life on prognosis was a secondary outcome of this study, patients with high or low quality of life groups could be inhomogeneous. In our population, overall median survival was 14 months, that is slightly shorter than median survival reported in previous studies [6]. The reasons underlying this difference can reside in the high symptom-burden of the patients enrolled in our study, but further specifically designed studies are necessary to assess prognosis in this selected population.

This study has several limitations. Particularly, a relatively small sample size was enrolled in a large timespan. This is mainly and luckily due to the rarity of the disease in our geographic area. Secondarily, undetected, and unmeasured bias may have operated in the selection criteria between procedures. As we stated, patients enrolled at the beginning of the study were more likely to undergo extrapleural pneumonectomy as it was the surgical treatment of choice at the time. During the study period, surgical treatment choice moved towards extended pleurectomy/decortication. However, no differences could be found between the two populations in terms of disease stage, performance status, or in the other preoperative measured characteristics.

Another limitation could be represented by the type of questionnaires used. In fact, we privileged the investigation of the general quality of life instead of an instrument more specific for neoplastic disease. SF-36 and SGRQ were administered to patients with any thoracic condition, considering them more appropriate to detect quality of life changes, especially in patients undergoing such an extensive pulmonary resection. Moreover, the chemotherapy regimen adopted during the study was mostly based on classic chemotherapeutic agents.

Finally, it was impossible to evaluate the impact of the recurrence on the quality of life due to the retrospective nature of this study and it was impossible to retrieve data about chemo/radiotherapy drop out during treatment.

## 5. Conclusions

In this study, we demonstrated that patients affected with high symptom-burden malignant pleural mesothelioma may benefit from surgery. In our experience, pleurectomy/decortication achieved some temporary advantages in symptom control and the quality of life, especially in the first months after surgery. We also noticed a more effective and long-lasting pain control after extrapleural pneumonectomy. Unexpectedly, this procedure was also able to achieve a more durable relief of dyspnea and to improve dynamic resistance to strain despite the immediate and obvious reduction of forced vital capacity. We can conclude that although cytoreductive surgery proved not very effective in prolonging overall survival in malignant pleural mesothelioma, it can have some effects on symptom control and the quality of life in patients with high symptom-burden. Hence, despite poor survival results and challenging efforts for both patients and surgeons, it remains a worthy and valid option in patients with a so limited chance of care.

## Figures and Tables

**Table 1:** Peri-operative characteristics of the enrolled population.

Variable	Total (*n* = 55)	PD (*n* = 26)	EPP (*n* = 29)	*p*-Value
Age, years ± SD	61.8 ± 10.0	63.0 ± 10.6	60.8± 9.4	0.41
Gender, *n* (%)				0.47
Male	49 (89.1%)	24 (92.3%)	25 (86.2%)	
Histology, *n* (%)				0.48
Epithelioid	37 (67.3%)	16 (61.5%)	21 (72.4%)	
Biphasic	11 (20.0%)	7 (26.9%)	4 (13.8%)	
Sarcomatous	7 (12.7%)	3 (11.6%)	4 (13.8%)	
N2 disease, *n* (%)	12 (21.8%)	5 (19.2%)	7 (24.1%)	0.66
Stage, *n* (%)				0.13
I	18 (32.7%)	6 (23.1%)	12 (41.4%)	
II	24 (43.6%)	15 (57.7%)	9 (31.0%)	
III	13 (23.6%)	5 (19.2%)	8 (27.6%)	
Chemotherapy, *n* (%)				0.45
Neoadjuvant	12 (21.8%)	4 (15.4%)	8 (27.6%)	
Adjuvant	40 (72.7%)	21 (80.8%)	19 (65.5%)	
Neo + adjuvant	3 (5.5%)	1 (3.8%)	2 (6.9%)	
Pre-operative dyspnea (mMRC), *n* (%)				0.48
0	0 (0.0%)	0 (0.0%)	0 (0.0%)	
1	4 (7.3%)	1 (3.8%)	3 (10.3%)	
2	9 (16.4%)	4 (15.4%)	5 (17.3%)	
3	20 (36.4%)	12 (46.2%)	8 (27.6%)	
4	22 (40.0%)	9 (34.6%)	13 (44.8%)	
Karnofski index, *n* (%)				0.96
100%	8 (14.5%)	4 (15.4%)	4 (13.8%)	
90%	27 (49.1%)	13 (50.0%)	14 (48.3%)	
80%	20 (36.4%)	9 (34.6%)	11 (37.9%)	
Pre-operative pain (VAS), *n* (%)				0.11
1–2	0 (0.0%)	0 (0.0%)	0 (0.0%)	
3	6 (10.9%)	2 (7.7%)	4 (13.8%)	
4	5 (9.1%)	3 (11.5%)	2 (6.9%)	
5	1 (1.8%)	1 (3.8%)	0 (0.0%)	
6	14 (25.5%)	10 (38.5%)	4 (13.8%)	
7	17 (30.9%)	4 (15.4%)	13 (44.8%)	
8	12 (21.8%)	6 (23.1%)	6 (20.7%)	
9–10	0 (0.0%)	0 (0.0%)	0 (0.0%)	
Follow-up, months ± SD	16.5 ± 10.4	15.4 ± 9.1	17.4 ± 11.5	0.47
Morbidity, *n*(%)	17 (30.9%)	5 (19.2%)	12 (41.4%)	0.076
90-day Post-operative mortality, *n* (%)	3 (5.4%)	1 (3.8%)	2 (6.9%)	0.62

EPP: extrapleural pneumonectomy; PD: pleurectomy/decortication; SD: standard deviation; VAS: visual analogue scale. mMRC: modified Medical Research Council.

**Table 2:** Inclusion and exclusion criteria for the two surgical approaches.

**Pleurectomy/decortication**
Karnofsky score ≤ 70
FEV1 ≤ 40% of the predicted value or ≤1 L
Contralateral hypoperfused lung (<55% of the right or 45% of the left) at ventilation/perfusion scan
Left ventricular function ≤ 45%; Pulmonary artery pressure < 50 mmHg
Localized neoplastic invasion of the lung or neoplastic foci depth from the visceral pleura ≤ 30 mm
**Extrapleural pneumonectomy**
Karnofsky score > 70
FEV1 > 40% of the predicted value or >1 L
Contralateral lung perfusion ≥ 55% for the right lung or ≥45% for the left lung at ventilation/perfusion scan
Left ventricular function > 45%; pulmonary artery pressure < 50 mmHg
Extensive neoplastic invasion of the lung or neoplastic foci depth from the visceral pleura > 30 mm
**Exclusion criteria for any surgical approach**
Disease involving the contralateral hemithorax; transdiaphragmatic, transpericardial and/or extensive chest wall involvement; clinical N2/N3 disease
Creatinine > 2 mg/dL
Aspartate aminotransferase > 80 UI/L, total bilirubin > 1.9 mg/dL, prothrombin time > 15 s

**Table 3:** Comparison of the measured symptoms and quality of life determinants at fixed time between extrapleural pneumonectomy (EPP) and pleurectomy/decortication (PD).

Variable		PRE-OP		*p*-Value	POST-3mos		*p*-Value	POST-6mos		*p*-Value	POST-12mos		*p*-Value	POST-24mos		*p*-Value
		PD (*n* = 26)	EPP (*n* = 29)		PD (*n* = 25)	EPP (*n* = 28)		PD (*n* = 22)	EPP (*n* = 22)		PD (*n* = 14)	EPP (*n* = 16)		PD (*n* = 5)	EPP (*n* = 9)	
Pain		6.1 ± 1.5	6.3 ± 1.7	0.38	4.7 ± 1.5	4.3 ± 1.3	0.30	4.3 ± 1.5	3.6 ± 1.3	0.081	5.8 ± 1.4	3.8 ± 2.4	0.005	6.0 ± 1.3	4.4 ± 1.5	0.073
6 min walk		380.8 ± 53.4	384.6 ± 61.2	0.86	430.2 ± 39.6	439.8 ± 42.8	0.31	420.0 ± 42.3	418.4 ± 46.8	0.99	418.3 ± 68.0	417.0 ± 29.1	0.28	390.0 ± 8.9	410.0 ± 34.2	0.18
Dyspnea index		3.1 ± 0.8	3.0 ± 1.1	0.84	2.1 ± 0.6	2.1 ± 0.6	0.89	2.2 ± 0.7	2.2 ± 0.6	0.68	2.5 ± 0.9	2.4 ± 0.7	0.76	3.3 ± 0.5	2.6 ± 0.7	0.088
Forced vital capacity		65.6 ± 10.0	70.8 ± 11.4	0.074	69.6 ± 10.6	63.8 ± 11.2	0.073	68.7 ± 9.8	63.5 ± 10.2	0.095	63.2 ± 9.8	56.8 ± 8.5	0.067	60.8 ± 8.0	47.8 ± 5.0	0.003
SF-36	Body pain	13.7 ± 20.9	14.1 ± 20.6	0.90	37.5 ± 14.6	37.9 ± 14.1	0.94	30.5 ± 17.9	31.4 ± 18.6	0.79	27.9 ± 18.4	28.8 ± 15.8	0.97	11.0 ± 18.0	16.0 ± 21.7	0.71
	Physical functioning	61.3 ± 20.2	60.6 ± 19.3	0.95	66.3 ± 19.7	66.5 ± 18.7	0.99	64.3 ± 15.7	67.0 ± 16.8	0.58	61.1 ± 23.9	66.3 ± 25.8	0.52	53.3 ± 27.7	62.5 ± 28.8	0.71
	Role-function physical	42.0 ± 30.4	40.2 ± 29.1	0.81	62.0 ± 28.1	62.5 ± 26.8	1.00	52.3 ± 29.8	55.0 ± 29.8	0.76	45.0 ± 40.3	51.5 ± 41.9	0.65	25.0 ± 31.6	37.5 ± 41.2	0.63
	General health	31.0 ± 31.8	31.1 ± 30.3	0.78	45.1 ± 23.1	47.0 ± 23.5	0.79	40.1 ± 19.9	44.8 ± 23.5	0.49	35.4 ± 27.7	39.3 ± 29.31	0.74	24.5 ± 21.8	31.4 ± 28.4	0.71
	Vitality	42.2 ± 15.4	41.8 ± 14.6	0.87	52.4 ± 17.2	52.0 ± 16.5	0.88	40.9 ± 22.7	44.0 ± 22.9	0.65	36.7 ± 19.7	38.8 ± 20.1	0.74	49.2 ± 25.0	44.0 ± 25.8	0.71
	Social functioning	62.5 ± 20.1	61.6 ± 19.2	0.91	67.5 ± 25.0	68.3 ± 23.9	0.93	56.8 ± 21.4	61.5 ± 23.9	0.50	54.2 ± 22.0	59.6 ± 24.4	0.50	52.1 ± 12.3	59.7 ± 22.3	0.69
	Role-function emotional	64.0 ± 49.0	63.1 ± 48.3	0.88	76.0 ± 36.7	78.6 ± 35.4	0.76	69.6 ± 27.0	73.3 ± 27.2	0.56	57.8 ± 44.5	60.8 ± 42.9	0.88	66.6 ± 36.5	63.3 ± 36.7	0.87
	Mental health	55.7 ± 23.6	55.7 ± 22.3	0.95	61.8 ± 26.7	61.4 ± 25.2	0.83	50.4 ± 33.5	54.7 ± 33.7	0.60	47.7 ± 26.8	56.2 ± 29.2	0.39	44.0 ± 25.3	55.2 ± 31.1	0.43
	Physical component summary	29.0 ± 11.7	28.4 ± 11.3	0.98	37.1 ± 10.5	37.1 ± 9.9	0.85	34.3 ± 10.2	34.6 ± 10.8	0.85	31.0 ± 14.0	33.3 ± 14.7	0.68	24.7 ± 14.4	28.5 ± 16.2	0.87
	Mental component summary	48.6 ± 8.3	48.7 ± 8.1	0.92	50.7 ± 7.8	50.7 ± 7.4	0.87	45.1 ± 12.0	45.6 ± 12.0	0.87	39.4 ± 15.3	41.9 ± 15.2	0.60	28.7 ± 19.0	27.3 ± 16.1	0.87
SGRQ	Symptoms	32.8 ± 26.4	32.5 ± 27.8	0.90	22.2 ± 31.8	22.5 ± 31.8	0.97	22.8 ± 25.1	23.1 ± 26.0	0.88	30.3 ± 27.4	29.3 ± 25.5	0.94	36.7 ± 36.9	34.2 ± 32.5	0.87
	Activity	44.7 ± 29.9	43.8 ± 29.6	0.90	33.0 ± 29.7	33.6 ± 30.9	0.99	32.0 ± 32.0	32.8 ± 33.0	0.99	39.8 ± 32.8	38.7 ± 29.1	1.00	46.0 ± 26.8	47.1 ± 22.7	1.00
	Impact	23.4 ± 16.5	22.9 ± 16.8	0.84	18.9 ± 15.6	19.0 ± 16.21	0.95	19.7 ± 18.5	18.6 ± 17.7	0.91	21.2 ± 19.3	21.2 ± 18.3	0.91	23.9 ± 17.2	25.3 ± 15.9	1.00
	Total	43.4 ± 30.0	42.4 ± 30.4	0.86	35.9 ± 29.7	35.9 ± 30.7	0.90	29.9 ± 30.8	30.3 ± 31.1	0.91	35.4 ± 29.5	35.3 ± 27.7	0.93	49.3 ± 40.3	51.5 ± 38.7	0.85

6 min walk: six minute walking test; EPP: extrapleural pneumonectomy; PD: pleurectomy decortication; pre-op: pre-operative; mos: months; SF-36: short form health survey-36; SGRQ: Saint George Respiratory Questionnaire.

**Table 4:** Comparison of the measured symptoms and quality of life determinants between preoperative values and values measured at fixed time spawn in patients undergoing pleurectomy/decortication.

Variable PD (N. Patients)	PRE-OP (*n* = 26)	POST-3mos (*n* = 25)	*p*-Value	POST-6mos (*n* = 22)	*p*-Value	POST-12mos (*n* = 14)	*p*-Value	POST-24mos (*n* = 5)	*p*-Value
Pain	6.1 ± 1.5	4.7 ± 1.5	0.013	4.3 ± 1.5	0.005	5.8 ± 1.4	0.19	6.0 ± 1.3	0.10
6 min walking test	380.8 ± 53.4	430.2 ± 39.6	0.002	420.0 ± 42.3	0.010	418.2 ± 68.0	0.032	390.0 ± 8.9	0.67
Dyspnea index	3.1 ± 0.8	2.1 ± 0.6	<0.001	2.2 ± 0.7	0.002	2.5 ± 0.9	0.094	3.3 ± 0.5	1.00
Forced vital capacity	65.6 ± 10.0	69.6 ± 10.6	0.002	68.7 ± 9.8	0.039	63.2 ± 9.8	0.13	60.8 ± 8.0	0.89
Bodily pain SF-36	13.7 ± 20.9	37.5 ± 14.6	<0.001	30.5 ± 17.9	0.011	27.9 ± 18.4	0.056	11.0 ± 18.0	0.41
Physical functioning SF-36	61.3 ± 20.2	66.3 ± 19.7	0.095	64.3 ± 15.7	0.16	61.1 ± 23.9	0.64	53.3 ± 27.7	0.92
Role-functioning physical SF-36	42.0 ± 30.4	62.0 ± 28.1	0.002	52.3 ± 29.8	0.068	45.0 ± 40.3	0.50	25.0 ± 31.6	1.00
General health SF-36	31.0 ± 31.8	45.1 ± 23.1	0.012	40.1 ± 19.9	0.019	35.4 ± 27.7	0.42	24.5 ± 21.8	0.53
Vitality SF-36	42.2 ± 15.4	52.4 ± 17.2	0.011	40.9 ± 22.7	0.86	36.7 ± 19.7	0.20	49.2 ± 25.0	0.27
Social functioning SF-36	62.5 ± 20.1	67.5 ± 25.0	0.12	56.8 ± 21.4	0.77	54.2 ± 22.0	0.64	52.1 ± 12.3	0.13
Role-functioning emotional SF-36	64.0 ± 49.0	76.0 ± 36.7	0.34	69.6 ± 27.0	0.57	57.8 ± 44.5	0.74	66.6 ± 36.5	0.22
Mental health SF-36	55.7 ± 23.6	61.8 ± 26.7	0.23	50.4 ± 33.5	0.54	47.7 ± 26.8	0.20	44.0 ± 25.3	0.60
Physical component summary	29.0 ± 11.7	37.1 ± 10.5	0.001	34.3 ± 10.2	0.036	31.0 ± 14.0	0.82	24.7 ± 14.4	0.75
Mental component summary	48.6 ± 8.3	50.7 ± 7.8	0.15	45.1 ± 12.0	0.32	39.4 ± 15.3	0.063	28.7 ± 19.0	0.026
Symptoms SGRQ	32.8 ± 26.4	22.2 ± 31.8	0.042	22.8 ± 25.1	0.053	30.3 ± 27.4	0.59	36.7 ± 36.9	0.89
Activity SGRQ	44.7 ± 29.9	33.0 ± 29.7	0.029	32.0 ± 32.0	0.007	39.8 ± 32.8	0.82	46.0 ± 26.8	0.46
Impact SGRQ	23.4 ± 16.5	18.9 ± 15.6	0.12	19.7 ± 18.5	0.44	21.2 ± 19.3	0.82	23.9 ± 17.2	0.92
Total SGRQ	43.4 ± 30.0	35.9 ± 29.7	0.12	29.9 ± 30.8	0.005	35.4 ± 29.5	0.099	49.3 ± 40.3	0.75

Mos: months; PD: pleurectomy/decortication; SF-36: short form health survey-36; SGRQ: Saint George Respiratory Questionnaire.

**Table 5:** Comparison of the measured symptoms and quality of life determinants between preoperative values and values measured at fixed time spawn in patients undergoing extrapleural pneumonectomy.

Variable EPP (N. Patients)	PRE-OP (*n* = 29)	POST-3mos (*n* = 28)	*p*-Value	POST-6mos (*n* = 22)	*p*-Value	POST-12mos (*n* = 16)	*p*-Value	POST-24mos (*n* = 9)	*p*-Value
Pain	6.3 ± 1.7	4.3 ± 1.3	0.001	3.6 ± 1.3	<0.001	3.8 ± 2.4	0.006	4.4 ± 1.5	0.013
6 min walking test	384.6 ± 61.2	439.8 ± 42.8	0.001	418.4 ± 46.8	0.006	417.0 ± 29.1	0.13	410.0 ± 34.2	0.021
Dyspnea index	3.0 ± 1.1	2.1 ± 0.6	0.003	2.2 ± 0.6	0.001	2.4 ± 0.7	0.002	2.6 ± 0.7	0.021
Forced vital capacity	70.8 ± 11.4	63.8 ± 11.2	0.001	63.5 ± 10.2	0.001	56.8 ± 8.5	0.001	47.8 ± 5.0	0.005
Bodily pain SF-36	14.1 ± 20.6	37.9 ± 14.1	<0.001	31.4 ± 18.6	0.008	28.8 ± 15.8	0.003	16.0 ± 21.7	0.17
Physical functioning SF-36	60.6 ± 19.3	66.5 ± 18.7	0.040	67.0 ± 16.8	0.042	66.3 ± 25.8	0.39	62.5 ± 28.8	0.51
Role-functioning physical SF-36	40.2 ± 29.1	62.5 ± 26.8	0.001	55.0 ± 29.8	0.021	51.5 ± 41.9	0.066	37.5 ± 41.2	0.39
General health SF-36	31.1 ± 30.3	47.0 ± 23.5	0.004	44.8 ± 23.5	0.006	39.3 ± 29.31	0.13	31.4 ± 28.4	0.57
Vitality SF-36	41.8 ± 14.6	52.0 ± 16.5	0.006	44.0 ± 22.9	0.54	38.8 ± 20.1	0.42	44.0 ± 25.8	0.52
Social functioning SF-36	61.6 ± 19.2	68.3 ± 23.9	0.044	61.5 ± 23.9	0.52	59.6 ± 24.4	0.69	59.7 ± 22.3	0.55
Role-functioning emotional SF-36	63.1 ± 48.3	78.6 ± 35.4	0.20	73.3 ± 27.2	0.35	60.8 ± 42.9	0.46	63.3 ± 36.7	0.11
Mental health SF-36	55.7 ± 22.3	61.4 ± 25.2	0.20	54.7 ± 33.7	0.97	56.2 ± 29.2	0.72	55.2 ± 31.1	0.88
Physical component summary	28.4 ± 11.3	37.1 ± 9.9	<0.001	34.6 ± 10.8	0.018	33.3 ± 14.7	0.24	28.5 ± 16.2	0.44
Mental component summary	48.7 ± 8.1	50.7 ± 7.4	0.18	45.6 ± 12.0	0.28	41.9 ± 15.2	0.15	27.3 ± 16.1	0.005
Symptoms SGRQ	32.5 ± 27.8	22.5 ± 31.8	0.033	23.1 ± 26.0	0.037	29.3 ± 25.5	0.60	34.2 ± 32.5	0.59
Activity SGRQ	43.8 ± 29.6	33.6 ± 30.9	0.049	32.8 ± 33.0	0.016	38.7 ± 29.1	0.83	47.1 ± 22.7	0.72
Impact SGRQ	22.9 ± 16.8	19.0 ± 16.21	0.20	18.6 ± 17.7	0.38	21.2 ± 18.3	0.87	25.3 ± 15.9	0.96
Total SGRQ	42.4 ± 30.4	35.9 ± 30.7	0.17	30.3 ± 31.1	0.005	35.3 ± 27.7	0.28	51.5 ± 38.7	0.57

EPP: extrapleural pneumonectomy; Mos: months; SF-36: short form health survey-36; SGRQ: Saint George Respiratory Questionnaire.
